# Appointment

**Published:** 1836

**Authors:** 


### VII.—Appointment.

John L. Riddell, M. D., late Adjunct Professor of Chemistry and Lecturer on Botany, in the Cincinnati College, has been appointed Professor of Chemistry in the Medical College of Louisiana; vice W. Byrd Powell, M. D., resigned. Dr. Riddell has entered on his duties.

